# Direct imaging of structural disordering and heterogeneous dynamics of fullerene molecular liquid

**DOI:** 10.1038/s41467-019-12320-4

**Published:** 2019-09-27

**Authors:** Jeongheon Choe, Yangjin Lee, Jungwon Park, Yunho Kim, Chae Un Kim, Kwanpyo Kim

**Affiliations:** 1 Department of Physics, Yonsei University, Seoul, 03722 Korea; 2 Department of Physics, Ulsan National Institute of Science and Technology (UNIST), Ulsan, 44919 Korea; 3 Center for Nanomedicine, Institute for Basic Science (IBS), Seoul, 03722 Korea; 4 School of Chemical and Biological Engineering, Institute of Chemical Process, Seoul National University, Seoul, 08826 Korea; 5 Center for Nanoparticle Research, Institute for Basic Science (IBS), Seoul, 08826 Korea; 6 Department of Mathematical Sciences, Ulsan National Institute of Science and Technology (UNIST), Ulsan, 44919 Korea

**Keywords:** Transmission electron microscopy, Structure of solids and liquids

## Abstract

Structural rearrangements govern the various properties of disordered systems and visualization of these dynamical processes can provide critical information on structural deformation and phase transformation of the systems. However, direct imaging of individual atoms or molecules in a disordered state is quite challenging. Here, we prepare a model molecular system of C_70_ molecules on graphene and directly visualize the structural and dynamical evolution using aberration-corrected transmission electron microscopy. E-beam irradiation stimulates dynamics of fullerene molecules, which results in the first-order like structural transformation from the molecular crystal to molecular liquid. The real-time tracking of individual molecules using an automatic molecular identification process elucidates the relaxation behavior of a stretched exponential functional form. Moreover, the directly observed heterogeneous dynamics bear similarity to the dynamical heterogeneity in supercooled liquids near the glass transition. Fullerenes on graphene can serve as a new model system, which allows investigation of molecular dynamics in disordered phases.

## Abstract (web summary)

Understanding the structural changes in disordered non-equilibrium materials is important for their processing, yet it remains experimentally challenging to follow the dynamics. Here, Choe et al. image the first-order like transition from crystal to liquid in a model molecular system adsorbed on graphene by tracking individual molecules in real time.

## Introduction

Disordered states of materials, such as supercooled liquid or glass, display peculiar non-equilibrium behavior and have been intensively investigated for decades^1–4^. Various glassy materials are widely used in industrial applications and consumer products, and a fundamental understanding of the disordered non-equilibrium structural phase may also facilitate advancement in material processing and fabrication. Structural rearrangements, which are often spatially heterogeneous, govern the various properties of glassy systems^2,4–6^. Yet, direct visualization of these dynamical processes has been difficult, and diffraction-based analysis such as structure factors or intermediate scattering functions has been mainly utilized only to yield the spatially averaged signals^1,2^. To complement this issue, researchers have utilized computer simulations^6,7^ or other macroscopic model systems such as granular^8^ or colloidal particles^9–11^, providing verification on spatially heterogeneous dynamics and increasing characteristic length scales near the glass transition.

The main experimental limitation in directly imaging atoms and molecules in disordered states can be overcome by modifying the sample geometry, to an atomically thin two-dimensional (2D) glassy system. Until now, 2D silica glass^12,13^, 2D carbon glass^14^, and Si atoms at the surface of amorphous Si^15^ have been successfully visualized using transmission electron microscopy (TEM) or scanning tunneling microscopy (STM). Although direct imaging of atomic structure has been successfully performed in these atomic disordered systems, in-depth observation and analysis of both structure and dynamics at the atomic or molecular resolution are still mainly lacking. In particular, the strong covalent bond in the previously-studied atomic glass leads to relatively slow structural evolution and dynamics under experimental conditions, limiting systematic studies on the dynamical behavior.

Here, we prepare a C_70_ molecular system on graphene, and directly visualize both the structural and dynamical evolution of the system at molecular resolution. The relatively weak van der Waals interaction between C_70_ molecules can be perturbed using an electron-beam (e-beam) during aberration-corrected transmission electron microscopy (acTEM), emulating the melting process of the molecular crystal. Our computerized method precisely identifies molecular positions in the disordered state, and the pair correlation functions of molecules clearly show the short-range liquid-like ordering. Time-dependent relaxation behaviors of the molecular structure are studied in-depth by van Hove correlation functions, which clearly shows the relationship between the local structure ordering and the dynamical behavior of the system. Real-time tracking of individual molecules also allows us to extract the spatially heterogeneous dynamics during the melting process. Our study demonstrates that fullerenes on graphene can serve as a new model system for investigation of super-cooled liquid and glass at molecular resolution.

## Results

### Preparation of C_70_ crystals on graphene

Fullerenes have relatively high robustness to e-beam under TEM imaging conditions compared to other organic molecules and can serve as model molecules^16–19^. During e-beam irradiation, energetic electrons can occasionally transfer significant momentum and energy to the fullerene molecules via elastic collisions, generating structural displacements and dynamical motions^20^. In particular, the weak van der Waals interaction between fullerene molecules can be easily perturbed with an e-beam, inducing strong dynamical behavior. Indeed, researchers have previously observed an e-beam induced molecular dynamics of C_60_ in one-dimensional confinement of carbon nanotube^21^. In our study, we chose C_70_ deposited on graphene as an ultra-thin model molecular system. Graphene has high electrical/thermal conductivity and can further reduce the structural damage to samples because of fast energy transfers^22–24^. The atomically flat surface of graphene facilitates the dynamical behavior of the molecular liquid under imaging conditions, allowing systematic study of its molecular structure and dynamics. Compared to C_60_ fullerene^16,18,25^, C_70_ has a slight anisotropy in its molecular shape, which may also contribute to the more pronounced molecular movement.

We deposited C_70_ molecules onto a graphene membrane by thermal evaporation to make a thin film (see methods section for detailed information)^18^. Using electron diffraction, we find that the nearest neighbour distance between molecules in the initial C_70_ film was 1.07 nm without any sign of anisotropy in terms of C_70_ orientation (Supplementary Fig. 1). The observed thin-film (10 nm thick) C_70_ crystal structure is consistent with the high-temperature crystal phase of a face centered cubic (fcc)^16^. The film also exhibits a uniform and well-ordered structure (Supplementary Fig. 1). For the main study on the molecular structure and dynamics of liquid-like state, C_70_ samples with 3~5 nm thickness (3~5 molecular layers) were used.

### Melting of C_70_ crystals by e-beam irradiation

**Fig. 1:**
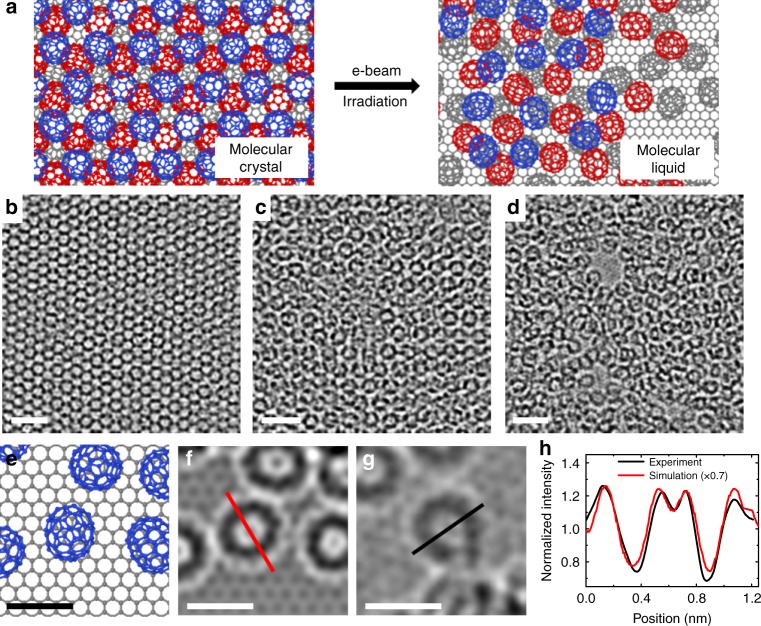
Electron beam irradiation on C_70_ molecular crystal. **a** Atomic models of C_70_ assembly on graphene showing the order-to-disorder structural transition under e-beam. **b**–**d** TEM images of C_70_ molecular arrangement under e-beam showing increased structural disorder. Scale bar, 2 nm. **e** Atomic model, **f** corresponding TEM simulation image, and **g** experimental TEM image of an isolated C_70_ molecule on graphene. Scale bar, 1 nm. **h** Intensity profile comparison between simulation and experimental images along red and black lines in **f**, **g**

A well-ordered C_70_ packing structure can be perturbed with e-beam irradiation (Fig. 1a). Figure 1b–d show TEM images of C_70_ film on graphene, revealing the structural transition from ordered to disordered configurations. E-beam irradiation drives the molecular crystal to a more disordered state, emulating the melting process of the molecular crystals. We note that the process is driven mainly through decreasing the molecular ordering by the random energy transfer of e-beam to molecules, not through actual heating of the system^13,14^. This raises the possibility that the effects of e-beam irradiation may not be the same as thermal effects. For example, in the case of single-layer graphene, it was shown that the population of e-beam induced defects deviates from a thermally induced Boltzmann distribution^26^. Nonetheless, e-beam irradiation can drive the system into high-energy states, similar to what can be expected in a thermally activated system. Such similarities have been studied theoretically in 2D silica^27^, and experimentally observed during the transformation of various graphitic nanostructures^28–30^.

As the C_70_ molecular long-range order diminishes under prolonged e-beam irradiation, we observed a change in the local C_70_ film thickness. The bare graphene surface, which can be regarded as a pore in the C_70_ film, was also observed locally as shown in Fig. 1d. The non-uniform film thickness under e-beam irradiation mainly results from the molecular rearrangements on the graphene surface rather than the molecular ejection because the energy barrier for the molecular migration is much lower than that for the molecular desorption from the surface.^20,31^ The molecular rearrangements seem to be driven by the stronger C_70_–C_70_ interactions than the C_70_–graphene interactions^31^. Around the pores in the C_70_ film, the local thickness of the film was sub-monolayer, and isolated C_70_ molecules with circular shapes could be clearly observed. The observed molecular shape (Fig. 1g) and the line intensity profile along the molecule (Fig. 1h) were consistent with the TEM simulation data (Fig. 1e, f).

**Fig. 2:**
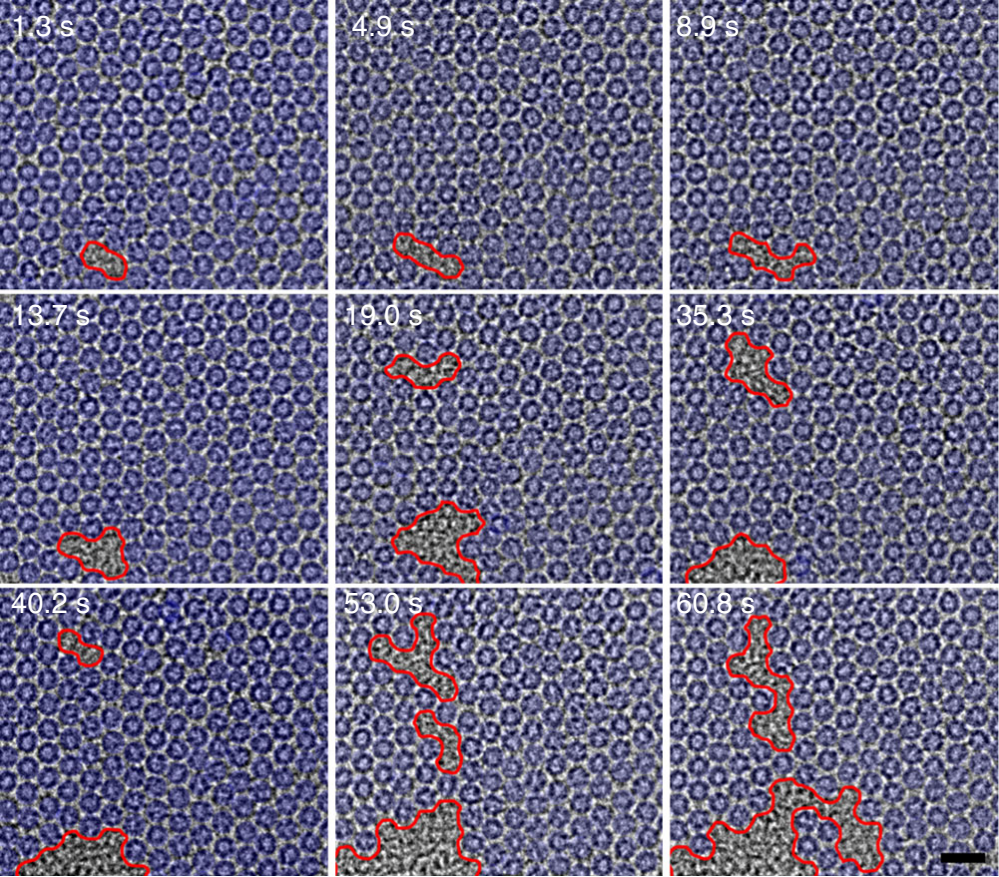
Initial melting from molecular crystal to liquid phase. A series of TEM images showing the initial structural transition from molecular crystal (pseudo-colored in blue) to liquid phase. The disordered molecular regions nucleate and gradually grow out of homogeneous crystalline phase with clear phase boundaries. Scale bar, 2 nm

Figure 2 is a series of TEM images showing the behavior of C_70_ molecular crystal at the very early stage of melting, namely stage 0. TEM images of C_70_ initially show highly-ordered molecular positions together with clearly visible circular molecular shape. Later, molecular movements were induced by e-beam irradiation, which results in local heterogeneous disordering of the molecules. The area bounded by red lines in Fig. 2 indicates the disordered molecular regions. We find that the disordered regions nucleated locally and were growing with fluctuations in their shape and eventually merging with adjacent regions over time as shown in Fig. 2 and Supplementary Movie 1. The nucleation and growth of a disordered phase out of a homogeneous crystalline phase and the observed microstructural phase coexistence with clear phase boundaries strongly suggest that the observed phase transition has the characteristics of a thermodynamic first-order phase transition^32,33^. This observation supports that the e-beam induced crystal-to-liquid melting in our study has similarity to the conventional crystal-to-liquid melting induced by thermal activation.

### Identification of C_70_ positions and pair correlation

The well-defined nearly circular shape of the C_70_ molecules, as shown by the dark circular line at our imaged condition (defocus value at −13 nm), can be used to efficiently identify molecular positions, even in an area where the direct recognition of molecules is difficult due to multiple molecular overlaps. To identify molecular positions reliably, we devised an image processing scheme whereby circles with a predesignated radius *r* were automatically identified. To achieve this, we processed TEM images through a two-phase Mumford–Shah (MS_2_) model^34^ and calculated the probability density function at each image pixel, PDF(*x*, *y*), which is a parameter indicating the probability of molecular presence at a certain pixel location (*x*, *y*). (See Supplementary Note 1 and Supplementary Fig. 2 for details). With a proper choice of PDF threshold, we could assign molecular positions in two-dimensional image space.

**Fig. 3:**
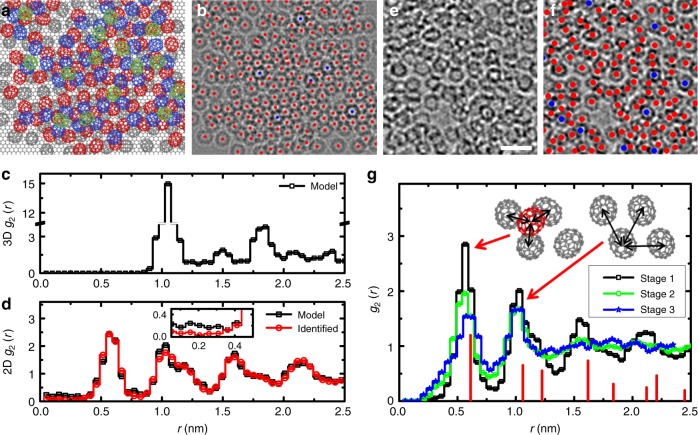
Identification of C_70_ positions in the disordered molecular structure. **a** Atomic model of disordered C_70_ structure on graphene. **b** Corresponding TEM simulation image of the model with identified center position overlay (red dots). Blue dots are doubly-counted positions. **c** 3D pair correlation function (PCF) from the disordered C_70_ molecular model. **d** 2D-projected PCF from the molecular model and identified positions. The inset shows the PCF at 0 ≤ *r* ≤ 0.5 nm. **e** Experimental TEM image of molecular liquid. Scale bar, 2 nm. **f** TEM image with identified center position overlay. **g** PCF of molecules from experimental data at different stages of melting. The red reference peaks indicate the calculated pair distances with ABC molecular stacking of C_70_ crystals. The inset schematics show the atomic models from ABC stacking

To validate our method, we first applied our image processing to simulated TEM images using a model with disordered molecules on graphene, as shown in Fig. 3a, b. The molecular model with ~3-nm thick fullerene film was constructed by Monte Carlo simulations, in which a reasonable three-dimensional (3D) molecular pair correlation was used as shown in Fig. 3c. The pair correlation function (PCF) is given by

1 $$g_2\left( r \right) = \frac{1}{{2{\mathrm{\pi }}rN\rho }}\mathop {\sum}\nolimits_{i,j\left( {i \ne j} \right)} {\delta \left( {r - r_{ij}} \right)}$$

where *r*_*ij*_ is the distance from the *i*th to the *j*th molecules, *N* is the total number of molecules, and *ρ* is the number density of molecules. The identification process was verified using simulation images with different noise levels (Supplementary Fig. 3). We found that the process identifies the centers of molecules with precision higher than 92% for a model with three molecular layers and the main inaccuracy of the identification process is originated from uncounted molecules due to significant molecular overlaps (Supplementary Fig. 3). The main effect of the uncounted molecules can be seen from the undercounted data points at *r* < 0.3 nm in 2D-projected PCF (2D *g*_2_(*r*)) as shown in the inset of Fig. 3d. Nevertheless, the general features in 2D *g*_2_(*r*), including the peak intensity and position, were well-captured by our identification process. The identification process was also validated with control images of amorphous carbon and computer-generated random noise (Supplementary Figs. 4 and 5). The well-known Hough transform^35,36^ and another scheme (simple circumference transform) were also tested, but we find that MS_2_ model yielded the most reliable identification of C_70_ molecules.

With the application of the MS_2_ model and PDF calculations, experimentally obtained TEM images were processed (Supplementary Movies 2–4) and the centers of molecules were identified, as shown in Fig. 3e, f. Figure 3g presents PCF at different stages of imaging (stage from 1 to 3), where the structural ordering was diminished as the molecular crystals underwent melting under e-beam irradiation. The PCF at its fully disordered state exhibited peaks around *r* *=* 0.5 and 1.0 nm and converged to unity in the range *r* *>* 2.0 nm. This observation clearly shows that the C_70_ molecular system hosts a liquid-like short-range ordering under e-beam irradiation. It is noticed that the peak positions in 2D PCF were slightly different from the expected positions (red lines) calculated from projection image using close-packed C_70_ fcc molecular assembly. In particular, the peak positions were slightly down-shifted, showing an apparent lattice contraction compared to the original C_70_ crystal due to 2D projection of 3D counter-part^37^. We note that the obtained PCF in Fig. 3g was calculated from 2D-projected TEM images, which shows some differences from the 3D PCF as shown in Fig. 3c, d. Tomography-based TEM imaging^38^ is potentially available to obtain a static molecular 3D PCF, but it is currently challenging to apply the technique to our dynamic molecular systems due to possible e-beam induced motions during extended experimental imaging time.

### Time-dependent correlation function of molecular structures

The detailed dynamical behaviors of C_70_ molecules can be studied using time-dependent correlation functions. Van Hove correlation functions (vHCF) were obtained using

2 $$G\left( {r,t} \right) = \frac{1}{N}\left\langle {\mathop {\sum}\nolimits_{i,j}^N {\delta ({\mathbf{r}} + {\mathbf{r}}_j\left( 0 \right) - {\mathbf{r}}_i\left( t \right))} } \right\rangle$$

**Fig. 4:**
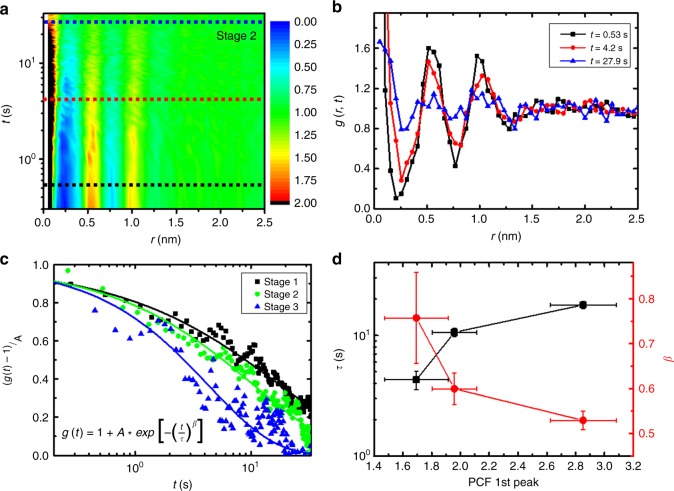
Time-dependent correlation functions of molecular structures. **a** van Hove correlation function (vHCF) of C_70_ molecular structure. **b** The evolution of vHCF. The plots are obtained at the dashed lines in **a**. **c** Time-evolution of the first peak (*r*~0.53 nm) in vHCF. **d** Relaxation time *τ* and decaying parameter *β* of the molecular structure as a function of structure order. The lateral error bars represent the standard deviations of measurements from 21 image frames. The vertical error bars represent the ranges of *τ* and *β* obtained from curve fitting to the data points in Fig. 4c with a stretched exponential function

Figure 4a shows *g*(*r,t*) = *G*(*r,t*)/*ρ* at the state where the disordered state was developed from the C_70_ crystal (stage 2). We note that the proper calculation of vHCF involves the overall drift correction of time-series TEM images, which is described in detail in Supplementary Fig. 6. The peaks at *r*~0.5 nm and *r*~1.0 nm show rapid decaying behaviors and converge to unity (Fig. 4b). The dynamical structural relaxation characteristics can be further investigated by analyzing the shape of the relaxation function. Figure 4c shows the decaying vHCF plots of the first peak (at *r*~0.5 nm) at the different stages of melting. The relaxation behavior can be fitted using $g\left( t \right) = 1 + A \ast {\mathrm{exp}}\left[ { - (t/\tau )^\beta } \right]$ with fitting parameters *τ* and *β*. The fitting functional form does a good job of capturing the experimental relaxation, resulting in approximately *τ*~10 s and *β*~0.6. The observed non-exponential decaying function with *β* < 1 (stretched exponential decaying) is a strong indicator of the heterogeneous dynamical behavior of the system^3–5,39^.

The observed dynamical relaxation characteristics exhibited correlations with local structure ordering. As shown in Fig. 4d, the relaxation time *τ* becomes smaller as the local structural order parameter, PCF 1^st^ peak, diminishes. In situations where the structural order parameter is high, the relaxation dynamics can be suppressed due to the higher energy barrier originated from the larger molecular coordination number, which can be obtained from ${\int}_0^{r = 1.1nm} {g_2\left( r \right)\left( {2{\mathrm{\pi }}r\rho } \right)dr}$. On the other hand, as the molecular structure undergoes disordering, the energy barrier for molecular movement can be lowered due to the smaller molecular coordination number. This result implies that the relaxation process is highly sensitive to local structural ordering and the observed heterogeneous dynamical behaviors can be originated from the heterogeneous structural ordering.

### Spatially resolved heterogeneous dynamics in C_70_ liquid

**Fig. 5:**
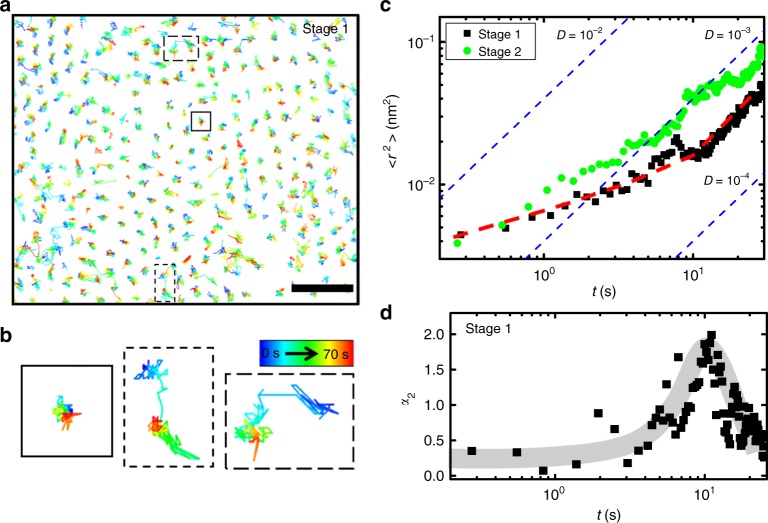
C_70_ dynamics in fullerene molecular liquid. **a** C_70_ molecular trajectories at the relatively ordered molecular packing structure. Scale bar, 2 nm. **b** Zoomed-in molecular trajectories from panel **a**, showing sporadic jumps. **c** Displacement behaviors of molecules at two different stages of observation. The red dashed line is a guide to the eye for stage 1 data. **d** Calculated non-Gaussian parameter *α*_2_ at stage 1. The gray area is a guide to the eye

The heterogeneous dynamical molecular behaviors can be further directly accessed by visualization of molecular movement during melting. With the ability to track dynamics at molecular resolution, we visualized the 2D diffusional behaviors of C_70_ molecules. Figure 5a shows 2D molecular trajectories of C_70_ molecules at a relatively early stage of melting (stage 1), demonstrating that the structural and dynamical behaviors of molecular disordering display spatially heterogeneous process. The central area of Fig. 5a shows a more ordered structure together with suppressed dynamical behavior, whereas the non-central area shows a more disordered structure with enhanced molecular dynamics. The positions of molecules with pronounced dynamics also display this heterogeneity, as shown in Supplementary Fig. 7. The zoomed-in molecular trajectories (Fig. 5b) show that the molecular diffusion is associated mainly with wiggling movements and sporadic large jumps. This non-trivial behavior is reminiscent of the cage rearrangement observed in model colloidal systems near the glass transition^10,11^.

Detailed diffusional behaviors of molecules can be examined using the mean-squared displacement of molecules defined by $\left\langle {r^2\left( t \right)} \right\rangle = \frac{1}{N}\mathop {\sum}\nolimits_{i = 1}^N {\left| {{\mathbf{r}}_i\left( t \right) - {\mathbf{r}}_i\left( 0 \right)} \right|^2}$, as shown in Fig. 5c. The mean-squared displacement at two different stages during the melting process clearly shows that the molecular diffusion became more pronounced at the later stage of observation. This is consistent with our analysis and conclusion from vHCF in Fig. 4. Interestingly, in Fig. 5c, the slope of the plots (red dashed line) begins with a lower value and then approaches the slope of the blue lines over time. The blue dashed lines indicate the diffusional behavior obtained from free diffusional form^6^. The time scale of the transition from sub-free to free diffusional behavior is ~10 s, which is consistent with the relaxation time scale observed in Fig. 4. Heterogeneous dynamical behavior can also be studied using the non-Gaussian parameter, $\alpha _2 = \left\langle {r^4\left( t \right)} \right\rangle /\left\langle {3r^2\left( t \right)} \right\rangle - 1,$ which quantifies deviations from a Gaussian distribution^5,10,11^. Calculated *α*_2_ at the stage 1 shows a peak value of around 2 at *t*~10 s as shown in Fig. 5d. The similar *α*_2_ feature showing a peak at the characteristic relaxation time scale was previously observed for the alpha-relaxation of various glass systems^5^. This supports that the structural rearrangements during melting display heterogeneous dynamics, and our molecular system bears some similarity to the dynamics observed in supercooled liquids near the glass transition^3,6^. We note that the effect of overall drift was eliminated by the drift correction during the post-imaging process as shown in Supplementary Fig. 6.

## Discussion

In conclusion, we observed an e-beam induced first-order transition-like crystal melting of C_70_ molecular system and analysed its molecular dynamics with single-molecule sensitivity. The pair correlations for C_70_ molecules were calculated using an automated molecular position identification process. The spatially heterogeneous dynamical behavior of these molecules bore the similarity to the dynamical heterogeneity observed in supercooled liquids near the glass transition. Considering the possibility of using various molecules with different anisotropy (C_60_, C_72_, and C_82_) and modifying interactions between molecules through surface/internal functionalization (C_60_F_48_ and M@C_82_)^16^, fullerenes on graphene can serve as a new model system for investigation of molecular glass and supercooled liquid, providing unprecedented real-space imaging of dynamical heterogeneity. Future work combining the e-beam irradiation with in situ heating will make it possible to reveal the details on the thermodynamic equilibrium and non-equilibrium properties of fullerene model systems.

## Methods

### Sample preparation

Graphene was synthesized using chemical vapor deposition (CVD)^40^. Twenty-five micrometer-thick copper foil was used as the synthesis substrate. CVD graphene was transferred to Quantifoil holey carbon grids via direct transfer^18^. C_70_ films were deposited onto graphene TEM grids by thermal evaporation. Before the thermal evaporation process, TEM grids were pre-annealed in the air at 200 °C for 30 min to minimize surface adsorbate on graphene. The C_70_ films with thicknesses ranging from 0.5 to 10 nm were deposited at the deposition rate of 0.05 Å s^−1^ under a vacuum pressure of 2 × 10^−6^ Torr. The graphene substrate was held at 110 °C during the deposition.

### TEM imaging and simulation

TEM imaging was performed using a FEI Titan equipped with an image aberration corrector operated at 80 kV and a JEOL ARM 200 F equipped with image and probe aberration correctors operated at 80 kV. Selected area electron diffraction was performed with a FEI Tecnai. The electron dose during TEM imaging was ~2 × 10^4^ e nm^−2^ s^−1^. TEM videos were recorded using the CamStudio program with a temporal resolution of around 0.2 s per frame. TEM image simulations were performed using MacTempas software with experimental imaging conditions. The simulation images were obtained at a defocus value of −13 nm.

### TEM image analysis

Molecular position identification was performed using the two-phase Mumford–Shah (MS_2_) model and the calculation of the probability density function (PDF) for molecular presence at each pixel. First, the MS_2_ model calculates the support function of an experimental TEM image. Based on the dark circular shape of fullerenes on the image, the annulus support with a given radius was applied to each image pixel. Second, the PDF was computed using the calculated support function and the chosen reference molecule, which described the probable molecular positions. To find positions that otherwise could be missed due to the aggregation of molecules, bright artificial molecules were inserted to the positions obtained from PDF, after which the PDF was re-calculated. Double-counted positions were determined if the PDF value was more than 98% of the PDF maximum. Drift correction of time-series TEM images was performed using a custom ImageJ macro. To quantify overall drift for each frame, tracking of molecular positions was executed for all frames, and the average displacement of the tracked molecules at a given frame was calculated along both the x and y directions. The overall drift was compensated using the calculated average displacement for a given frame. These processes are described in greater detail in the Supplementary Information section.

## Supplementary information

Supplementary Information

Description of Additional Supplementary Files

Supplementary Movie 1

Supplementary Movie 2

Supplementary Movie 3

Supplementary Movie 4

## Data Availability

The authors declare that the data supporting the findings of this study are available within the Supplementary Information files and from the corresponding authors upon reasonable request.

## Code availability

The codes used for data are available from the corresponding authors on reasonable request.

## References

[1] Zallen, R. *The Physics of Amorphous Solids*. (WILEY-VCH Verlag GmbH & Co. KGaA, 2004).

[2] Binder, K. & Kob, W. *Glassy Materials and Disordered Solids: An Introduction to Their Statistical Mechanics*. (World Scientific, 2011).

[3] Debenedetti PG, Stillinger FH (2001). Supercooled liquids and the glass transition. Nature.

[4] Richert R (2002). Heterogeneous dynamics in liquids: fluctuations in space and time. J. Phys. Condens. Matter.

[5] Ngai, K. L. *Relaxation and Diffusion in Complex Systems*. (Springer, 2011).

[6] Berthier L, Biroli G (2011). Theoretical perspective on the glass transition and amorphous materials. Rev. Mod. Phys..

[7] Flenner E, Szamel G (2015). Fundamental differences between glassy dynamics in two and three dimensions. Nat. Commun..

[8] Marty G, Dauchot O (2005). Subdiffusion and cage effect in a sheared granular material. Phys. Rev. Lett..

[9] Kegel WK, van Blaaderen A (2000). Direct observation of dynamical heterogeneities in colloidal hard-sphere suspensions. Science.

[10] Weeks ER, Crocker JC, Levitt AC, Schofield A, Weitz DA (2000). Three-dimensional direct imaging of structural relaxation near the colloidal glass transition. Science.

[11] Weeks ER, Weitz DA (2002). Properties of cage rearrangements observed near the colloidal glass transition. Phys. Rev. Lett..

[12] Lichtenstein L (2012). The atomic structure of a metal-supported vitreous thin silica film. Angew. Chem., Int. Ed..

[13] Huang PY (2013). Imaging atomic rearrangements in two-dimensional silica glass: watching silica’s dance. Science.

[14] Eder FR, Kotakoski J, Kaiser U, Meyer JC (2014). A journey from order to disorder—atom by atom transformation from graphene to a 2D carbon glass. Sci. Rep..

[15] Ashtekar S, Scott G, Lyding J, Gruebele M (2011). Direct imaging of two-state dynamics on the amorphous silicon surface. Phys. Rev. Lett..

[16] Dresselhaus, M. S., Dresselhaus, G. & Eklund, P. C. *Science of Fullerenes and Carbon Nanotubes: Their Properties and Applications*. (Elsevier Science, 1996).

[17] Tizei LHG (2014). Single molecular spectroscopy: identification of individual fullerene molecules. Phys. Rev. Lett..

[18] Kim K (2015). Structural and electrical investigation of C_60_–graphene vertical heterostructures. ACS Nano.

[19] Nakamura E (2017). Atomic-resolution transmission electron microscopic movies for study of organic molecules, assemblies, and reactions: the first 10 years of development. Acc. Chem. Res..

[20] Egerton RF (2013). Beam-induced motion of adatoms in the transmission electron microscope. Microsc. Microanal..

[21] Ran K, Zuo J-M, Chen Q, Shi Z (2011). Electron beam stimulated molecular motions. ACS Nano.

[22] Zan R (2013). Control of radiation damage in MoS_2_ by graphene encapsulation. ACS Nano.

[23] Algara-Siller G, Kurasch S, Sedighi M, Lehtinen O, Kaiser U (2013). The pristine atomic structure of MoS_2_ monolayer protected from electron radiation damage by graphene. Appl. Phys. Lett..

[24] Choe J (2016). Direct imaging of rotating molecules anchored on graphene. Nanoscale.

[25] Mirzayev R (2017). Buckyball sandwiches. Sci. Adv..

[26] Börner P, Kaiser U, Lehtinen O (2016). Evidence against a universal electron-beam-induced virtual temperature in graphene. Phys. Rev. B.

[27] Roy PK, Heuer A (2019). Ring statistics in 2D silica: effective temperatures in equilibrium. Phys. Rev. Lett..

[28] Kurasch S (2012). Atom-by-atom observation of grain boundary migration in graphene. Nano Lett..

[29] Chuvilin A, Kaiser U, Bichoutskaia E, Besley NA, Khlobystov AN (2010). Direct transformation of graphene to fullerene. Nat. Chem..

[30] Chuvilin A (2011). Self-assembly of a sulphur-terminated graphene nanoribbon within a single-walled carbon nanotube. Nat. Mater..

[31] Rey C, García-Rodeja J, Gallego LJ, Alonso JA (1997). Clusters and layers of C_60_ molecules supported on a graphite substrate. Phys. Rev. B.

[32] Jaeger G (1998). The ehrenfest classification of phase transitions: introduction and evolution. Arch. Hist. Exact. Sci..

[33] Peng Y, Wang Z, Alsayed AM, Yodh AG, Han Y (2010). Melting of colloidal crystal films. Phys. Rev. Lett..

[34] Kim Y, Tagare HD (2014). Intensity nonuniformity correction for brain MR images with known voxel classes. SIAM J. Imaging Sci..

[35] Illingworth J, Kittler J (1988). A survey of the hough transform. Comput. Vis., Gr. Image Process..

[36] Yuen HK, Princen J, Illingworth J, Kittler J (1990). Comparative study of hough transform methods for circle finding. Image Vis. Comput.

[37] Mountjoy G (1999). Order in two-dimensional projections of thin amorphous three-dimensional structures. J. Phys. Condens. Matter.

[38] Xu R (2015). Three-dimensional coordinates of individual atoms in materials revealed by electron tomography. Nat. Mater..

[39] Vidal Russell E, Israeloff NE (2000). Direct observation of molecular cooperativity near the glass transition. Nature.

[40] Li X (2009). Large-area synthesis of high-quality and uniform graphene films on copper foils. Science.

