# Author Correction: Direct imaging of structural disordering and heterogeneous dynamics of fullerene molecular liquid

**DOI:** 10.1038/s41467-019-13947-z

**Published:** 2020-01-14

**Authors:** Jeongheon Choe, Yangjin Lee, Jungwon Park, Yunho Kim, Chae Un Kim, Kwanpyo Kim

**Affiliations:** 1 Department of Physics, Yonsei University, Seoul, 03722 Korea; 2 Department of Physics, Ulsan National Institute of Science and Technology (UNIST), Ulsan, 44919 Korea; 3 Center for Nanomedicine, Institute for Basic Science (IBS), Seoul, 03722 Korea; 4 School of Chemical and Biological Engineering, Institute of Chemical Process, Seoul National University, Seoul, 08826 Korea; 5 Center for Nanoparticle Research, Institute for Basic Science (IBS), Seoul, 08826 Korea; 6 Department of Mathematical Sciences, Ulsan National Institute of Science and Technology (UNIST), Ulsan, 44919 Korea

**Keywords:** Transmission electron microscopy, Structure of solids and liquids

Correction to: *Nature Communications* 10.1038/s41467-019-12320-4, published online 27 September 2019.

The original version of this Article contained an error in the Acknowledgements, which was previously incorrectly given as ‘This work was mainly supported by the Basic Science Research Program through the National Research Foundation of Korea (NRF-2017R1A5A1014862 and NRF-2019R1C1C1003643) and grants from the Institute for Basic Science (IBS-R006-D1)’. The correct version states ‘IBS-R026-D1’ in place of ‘IBS-R006-D1’. This has been corrected in both the PDF and HTML versions of the Article.

