# Supplementary material for: Direct imaging of structural disordering and heterogeneous dynamics of fullerene molecular liquid
Source: Nat Commun. 2019 Sep 27;10:4395. doi: 10.1038/s41467-019-12320-4 (PMC6765016; doi:10.1038/s41467-019-12320-4)
Supplement: Supplementary file 1 — Supplementary Information [file 41467_2019_12320_MOESM1_ESM.pdf]

Supplementary Information for

Direct imaging of structural disordering and heterogeneous dynamics of fullerene molecular liquid

Jeongheon Choe et al.

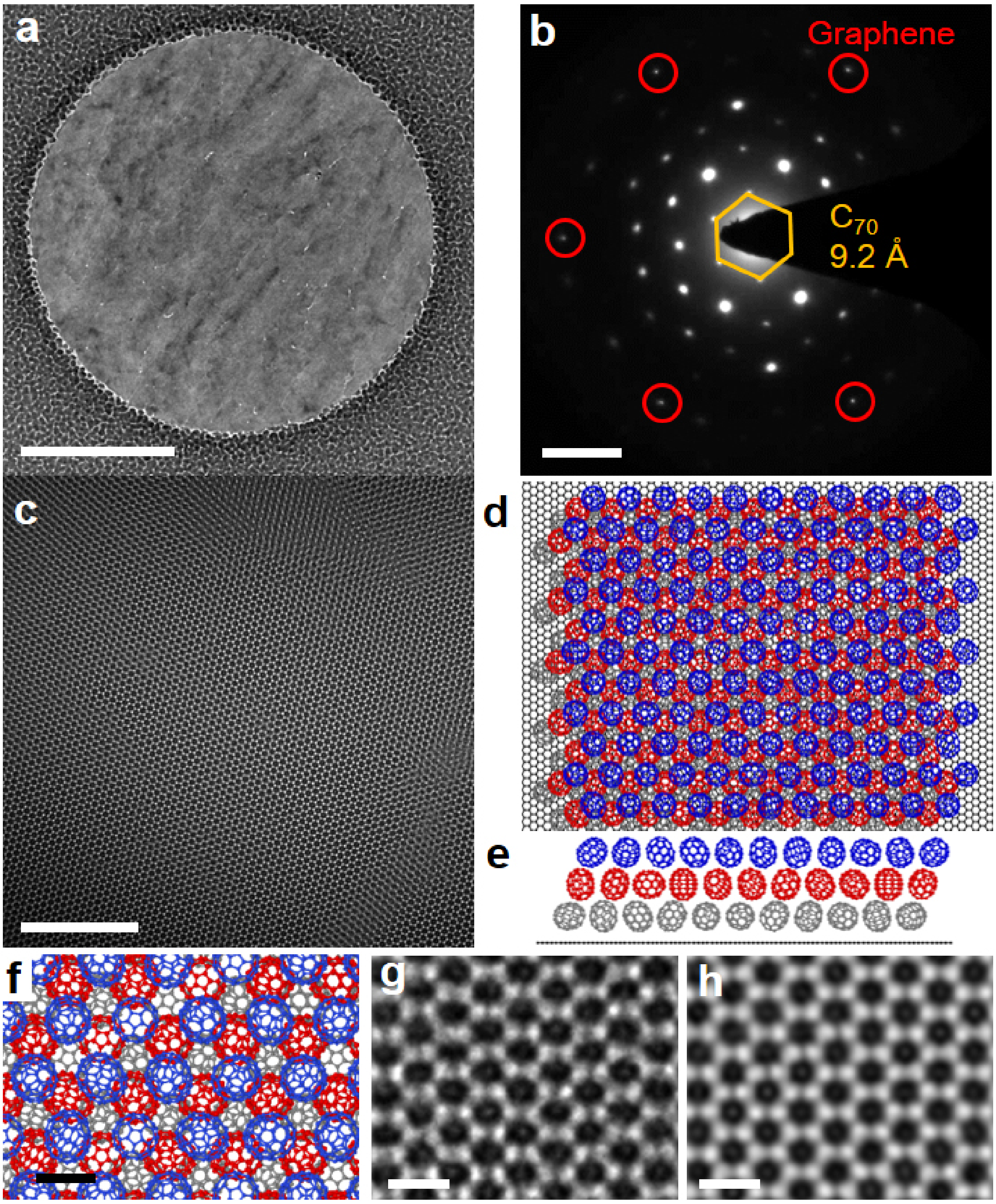

Supplementary Figure 1. TEM analysis of C_{70} crystal on a graphene substrate. (a) Bright-field TEM image of C_{70} crystal on a graphene TEM grid. Scale bar, 1 μm. (b) Electron diffraction of C_{70} crystal on graphene. Scale bar, 2 nm^{-1}. (c) HRTEM image of the sample. A uniform, well-ordered C_{70} crystalline arrangement is observed. Scale bar, 10 nm. (d-e) ABC stacking model of C_{70} on graphene (top and side view). (f) Zoomed-in atomic model, (g) TEM image and (h) simulated image of the crystal (defocus value at 15 nm). Scale bar, 1 nm.

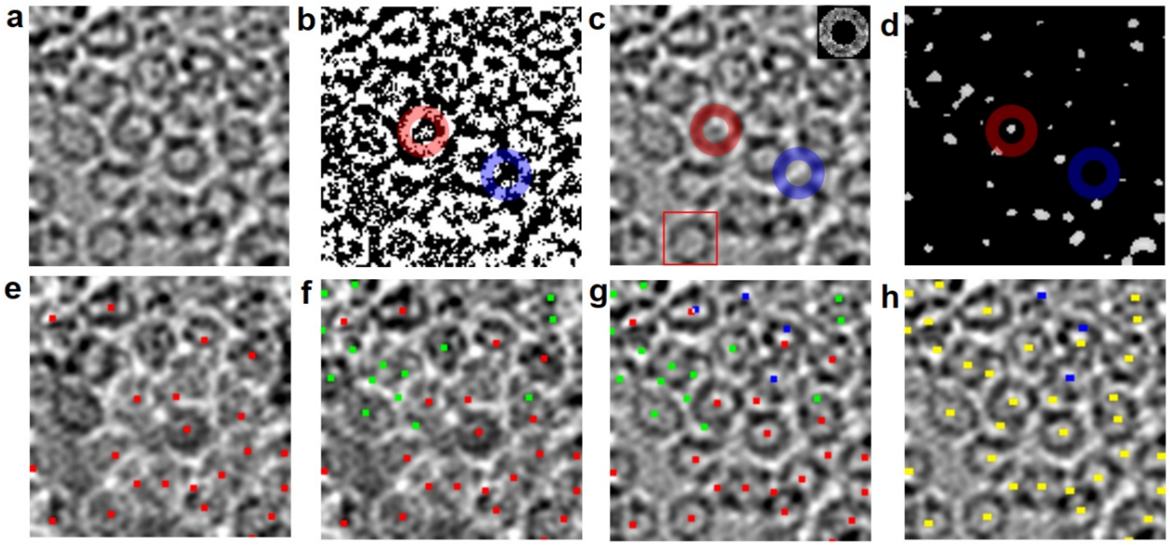

Supplementary Figure 2. Molecular center identification process. (a-d) Calculation of support function and the first probability density function (PDF). (a) Experimental TEM image. (b) Computed support function for molecules using the mathematical model (MS_2). The image is expressed in terms of a binary form. Red annulus support represents the highly probable position of a fullerene, and the blue one is vice versa. (c) The red square denotes the chosen reference molecule. The inset shows the reference molecule in the annulus support. (d) Truncated PDF, i.e., the probability of a molecule's presence at the pixel. The PDF is proportional to the number of white pixels in support of (b) and the ratio of the average pixel value in support of (c). (e-f) Insertion of artificial molecules into the experimental image to compute the PDF. The colored dots are from the centers from the first PDF (red dots) and the second PDF (green dots). The bright artificial molecules are added to the corresponding set of molecular centers. (g-h) Center decision process. (g) TEM image with the identified center position overlay. The blue points are from the third set of centers found using the third PDF. (h) This represents the final decision for all the centers. The yellow points are those chosen from the two sets of centers, red and green in (f), using the first PDF, while the green points are chosen from the third set of centers in (g) using the first PDF.

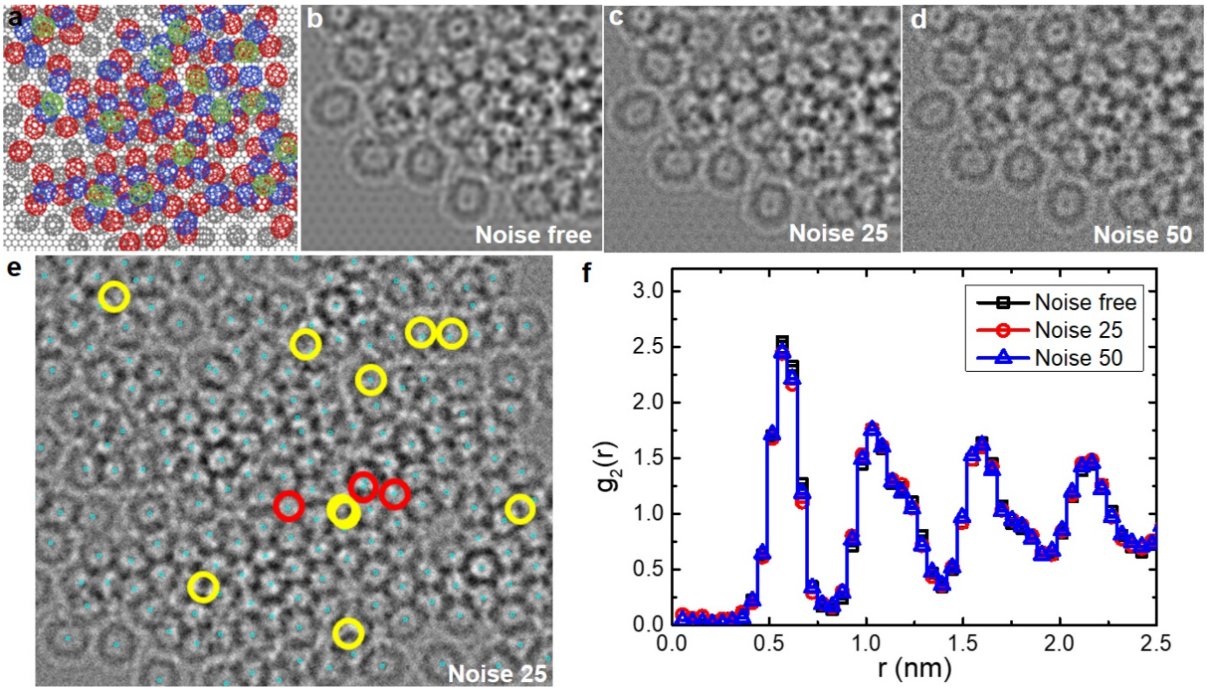

Supplementary Figure 3. Molecular center identification using TEM simulation images from a molecular model. (a) Atomic model of disordered C_{70} structure on graphene. (b-d) TEM simulation images of the model using different noise levels. (b) Original simulation TEM image. (c) TEM image after noise addition of a noise level (25% of the standard deviation of the original image). (d) TEM image with a noise level (50%). (e) Identified molecular positions using the simulation image (noise level, 25%). The cyan dots are the identified molecular positions. The yellow circles indicate molecular positions from the model, which remains unidentified from the process. The red circles indicate misidentified positions (no molecule from the model). (f) The calculated PCF using identified molecular positions from different noise levels.

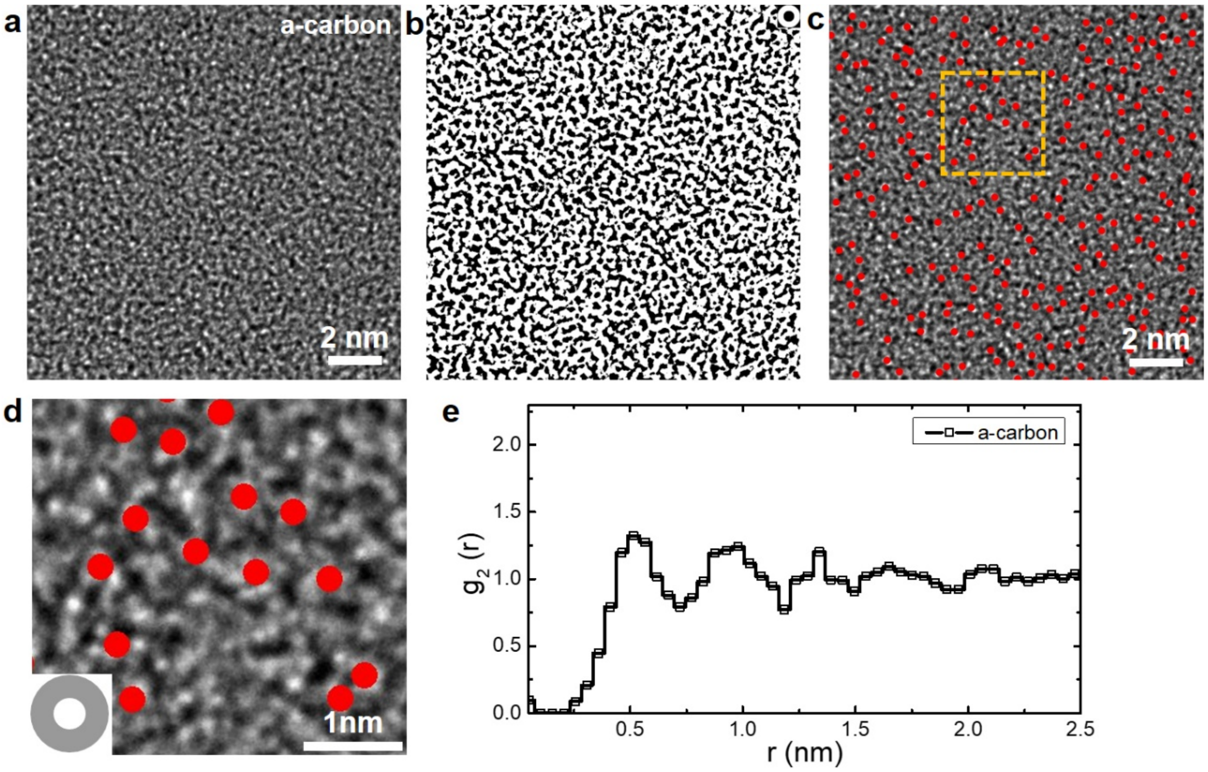

Supplementary Figure 4. Molecule identification process using an amorphous carbon image. (a) TEM image of 10-nanometer-thick amorphous carbon used for the center identification process. (b) Computed support function using the mathematical model (MS_2). The inset at the top-right corner shows a reference molecule shape. (c) TEM image with identified molecular position overlay (red dots). The dashed square is the field of view for panel d. (d) Zoomed-in TEM image with identified molecular position overlay. The inset at the bottom-left corner shows the reference molecule in the annulus support. (e) The calculated PCF using identified molecular positions.

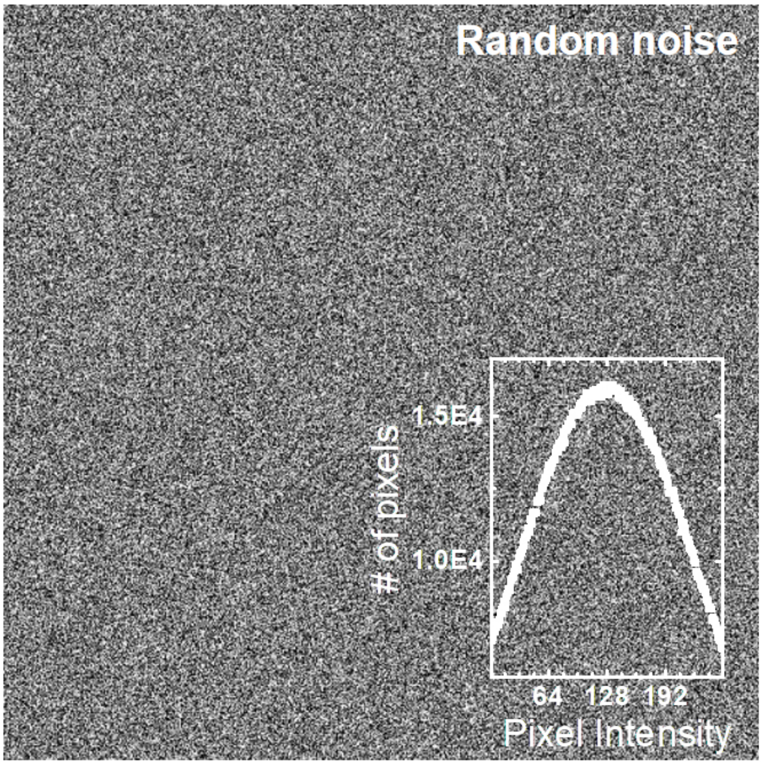

Supplementary Figure 5. Molecule identification trial using a computer-generated random noise. Computer-generated random noise image used for the center identification process. The process returned no identified molecules. The inset shows the histogram for the pixel intensity of the used random noise image.

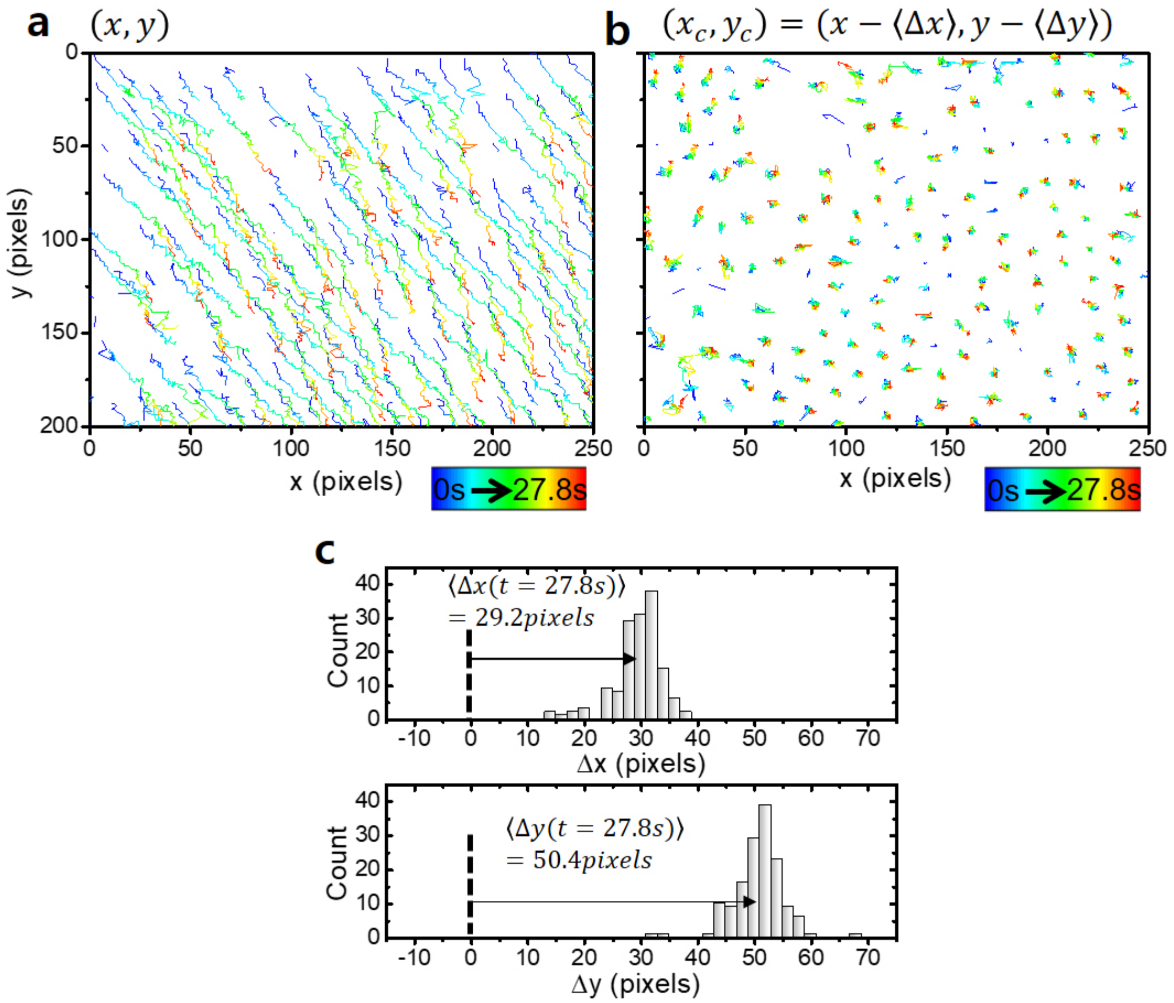

Supplementary Figure 6. Drift correction of time-series TEM images. Tracking molecular trajectories before (a) and after drift correction (b). (c) Histograms of x, y displacement of tracked molecules at t = 27.8 sec.

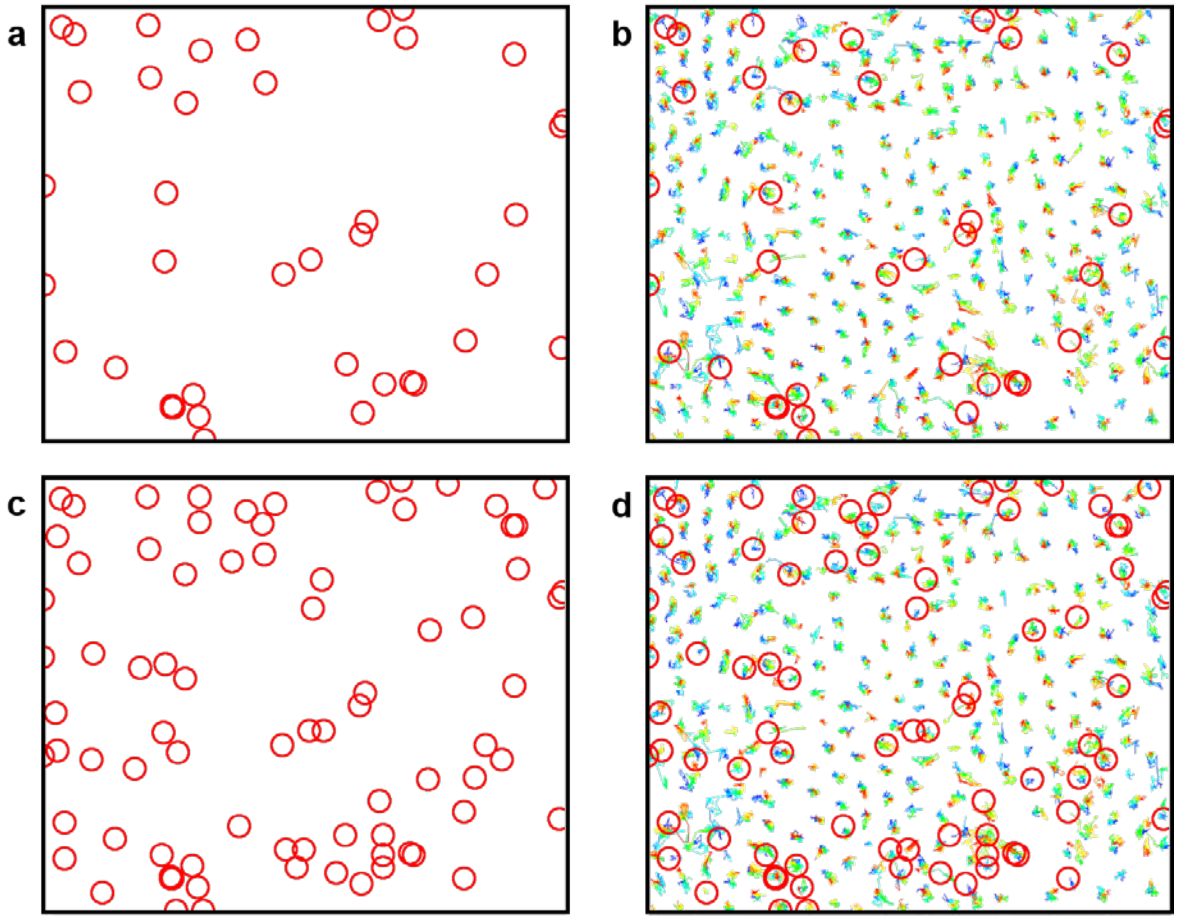

Supplementary Figure 7. The spatial distribution of fast-moving molecules. The initial positions of C_{70} molecules with the highest average speed within the top 10% (a) are shown as red circles. (b) Panel a with molecular trajectory overlay. (c) Molecular positions with top 20% highest speed (d) with their corresponding trajectory overlay.

Supplementary Note 1. Image processing for molecular center identification

For identification of the molecular centers in an experimental TEM image, we first estimated the regions where molecules are expected to exist via the 2-phase Mumford-Shah model (MS_2), shown below:

$$\min_{\substack{c_1,c_2\in\mathbb{R},\\ \Sigma\subset\Omega}} \operatorname{Per}(\Sigma;\Omega) + \lambda\Big(\int_\Sigma (f(x)-c_1)^2dx + \int_{\Omega\setminus\Sigma}(f(x)-c_2)^2dx\Big)$$

where Σ is the computed minimal solution, Ω is the whole image domain, f is the pixel value, x is the pixel position, and c_1 and c_2 are the corresponding pixel average of a binary function. Due to the nonlinear and nonconvex nature of the above MS_2 model, it is not easy to solve. However, one can modify the MS_2 model above for better analytical interpretation and numerical computation.[1] Indeed, by setting the following supplementary equation 1,

$$h_{c_1,c_2}(x) = (f(x)-c_1)^2 - (f(x)-c_2)^2 \qquad \text{(Supplementary eq. 1)}$$

one may view MS_2 as

$$\min_{\substack{c_1,c_2\in\mathbb{R},\\ \Sigma\subset\Omega}} \left(\left[\operatorname{Per}(\Sigma;\Omega) + \lambda\int_\Sigma h_{c_1,c_2}(x)dx\right] + \int_\Omega (f(x)-c_2)^2dx\right)$$

where the last term does not make any contribution to finding the region Σ, once c_1 and c_2 are estimated. Even though the problem still seems nonconvex, it turns out that this can be solved by a convex problem, even by a strongly convex problem with efficient and guaranteed algorithms. Supplementary Figure 2b shows the computed minimal solution Σ, which we define as the support of the experimental image. Thus, the support Σ of the image is the region where the centers of all molecules in the image are expected to reside.

Next, from among the experimental images we chose and fixed one reference molecule that possesses the standard shape and size (Supplementary Figure 2c). With the annulus support of the chosen molecule estimated, we computed the average pixel value in its support, which was robust to noise. (Inset of Supplementary Figure 2c) For each experimental image, we defined a probability density function (PDF) on the image domain, which presents the probability of each pixel point being a molecule's center (Supplementary Figure 2d). This PDF depends on two measures. One measure is the degree of overlap between the support of the image Σ and the annulus support of a molecule when its center sits at each image pixel. The other measure

is the ratio of the average pixel value in the annulus support of the reference molecule to the average pixel value in the annulus support of the same shape and size centered at each pixel point in the experimental image. These two measures are complementary to each other and enable us to define PDFs for all the experimental images.

To make our process more reliable, we computed three PDFs for each experimental image. The first PDF helps to find a set of possible centers, mainly in regions where molecules possess visually clear shapes. Once the first PDF was calculated, we inserted artificial molecules of the same shape and size as the reference molecule to the experimental image at the centers estimated from the first PDF (Supplementary Figure 2e). Then, we computed the second PDF with this modified experimental image, which finds another set of centers. Due to the previous procedure of artificial molecular insertion, the second set of centers that differs from the first set may explain where overlapped molecules with very close centers can exist. Collecting all the centers from the two sets, using the first PDF, we decided which ones are more likely to be true centers. Finally, we inserted artificial molecules again with the centers obtained by comparing the two previous sets to the unmodified experimental image (Supplementary Figure 2f) and computed the third PDF to avoid apparently missing centers (Supplementary Figure 2g). We used the first PDF again for the final decision (Supplementary Figure 2h). All the final decisions were made based on the first PDFs obtained from the original experimental images. This procedure was repeated for all videos.

One interesting feature of our scheme is that we can identify where more than one molecule can be overlapped with almost the same centers (Figure 3b and 3f). This was possible because we use more than one PDF for each experimental image to determine centers. If centers appear close to each other when using the first and the second PDFs, then they can be considered to be centers for overlapped molecules. We can also see slight differences in color in Supplementary Figure 2g, further suggesting molecular overlap.

Supplementary Note 2. Tracking of individual molecules

The molecular tracking was performed using a custom ImageJ macro. To find a realistic trajectory of each molecule, possible locations in the n+1th frame were examined around a target molecule of nth frame within a radius of 0.35 nm, which is less than the nearest neighbor distance ~ 0.5 nm. Among molecular location candidates, the nearest position of the n+1th frame was selected for updating its trajectory. For a forbidden location at the n+1th frame on tracking, the n+1th location was set as the middle point between the nth and n+2th frames. This procedure was applied to all molecular locations. After the tracking process, we executed an additional scheme linking the possible disconnections in all trajectories.

Supplementary Note 3. Disordered C_{70} structure modeling

We constructed an atomic model of disordered C_{70} layers on graphene using the Monte-Carlo method. To build a reasonable structure, the nearest neighbor distance of a C_{70} crystal was set as a constraint of a minimum inter-molecular distance. The center positions of the C_{70} molecules within a given layer were randomly assigned using the Monte-Carlo method with the constraint. The molecular orientation for the model was also randomly assigned (Figure 3a).

Supplementary References

(1) Kim, Y.; Tagare, H. D. Intensity Nonuniformity Correction for Brain MR Images with Known Voxel Classes. *SIAM J. Imaging Sci.* **2014,** *7*, 528-557.
