# Supplementary material for: Direct imaging of structural disordering and heterogeneous dynamics of fullerene molecular liquid
Source: Nat Commun. 2019 Sep 27;10:4395. doi: 10.1038/s41467-019-12320-4 (PMC6765016; doi:10.1038/s41467-019-12320-4)
Supplement: Supplementary file 2 — Description of Additional Supplementary Files [file 41467_2019_12320_MOESM2_ESM.pdf]

Description of Additional Supplementary Files

Supplementary Movie 1.
TEM video of melting C_{70} molecular crystal at the initial stage of melting. This video has a frame rate of 4.0 images per s.

Supplementary Movie 2.
TEM video of C_{70} molecules on graphene with identified molecular overlay at stage 1. This video has a frame rate of 3.6 images per s.

Supplementary Movie 3.
TEM video of C_{70} molecules on graphene with identified molecular overlay at stage 2. This video has a frame rate of 3.9 images per s.

Supplementary Movie 4.
TEM video of C_{70} molecules on graphene with identified molecular overlay at stage 3. This video has a frame rate of 4.4 images per s.
